# Perception Versus Reality: Subjective and Objective Neighborhood Characteristics and Cognitive Function in REGARDS

**DOI:** 10.1093/geroni/igaa057.2470

**Published:** 2020-12-16

**Authors:** Jessica Finlay, Philippa Clarke, Lisa Barnes

## Abstract

Does the world shrink as we age? The neighborhood captures a spatial area someone inhabits and moves through on a daily basis. It reflects a balance between internal perceptions and abilities, and the external environment which may enable or restrict participation in everyday life. We frequently hear that older adults have shrinking neighborhoods given declining functional mobility. This is associated with declines in physical and cognitive functioning, depression, poorer quality of life, and mortality. Knowledge of the interplay between objective and subjective neighborhood measurement remains limited. This symposium will explore these linked yet distinct constructs based on secondary data analyses of the Reasons for Geographic and Racial Differences in Stroke (REGARDS) study, a racially diverse sample of 30,000+ aging Americans. Finlay investigates how someone’s perceived neighborhood size (in number of blocks) varies by individual and geographic characteristics including age, cognitive function, self-rated health, and urban/rural context. Esposito’s analyses focus on neighborhood size in relation to race and residential segregation. Clarke compares subjective perceptions of neighborhood parks and safety from crime to objective indicators, and examines variations by health and cognitive status. Barnes will critically consider implications for how older adults interpret and engage with their surrounding environments. The symposium questions the validity of neighborhood-based metrics to reflect the perspectives and experiences of older residents, particularly those navigating cognitive decline. It informs policy-making efforts to improve physical neighborhood environments and social community contexts, which are critical to the health and well-being of older adults aging in place.

